# Supplementary material for: Platelet-Derived Growth Factor Induces SASP-Associated Gene Expression in Human Multipotent Mesenchymal Stromal Cells but Does Not Promote Cell Senescence
Source: Biomedicines. 2021 Sep 22;9(10):1290. doi: 10.3390/biomedicines9101290 (PMC8533296; doi:10.3390/biomedicines9101290)
Supplement: Supplementary file 1 [file biomedicines-09-01290-s001.zip › biomedicines-1353945-supplementary.pdf]

Supplementary
Fold change calculation

We calculated fold changes of gene expression relative to the expression in control sample:

$$FC_j = \begin{cases} 2^{e_j - c_j}, if e_j \geq c_j \\ -1 \times 2^{c_j - e_j}, if e_j < c_j \end{cases}$$

for each probe j (e denotes expression signal in experiment sample, c – signal in control sample).

Distributions of signal log ratios ($LR_j = e_j - c_j$) for 2 experimental samples are shown in figure 1.

We can see that changes in expression are not large (only few genes show twofold expression changes, corresponding to the log ratio of 1).
The threshold of 2 was chosen as more appropriate if we aimed to determine biological processes and pathways preferentially affected by treatment.

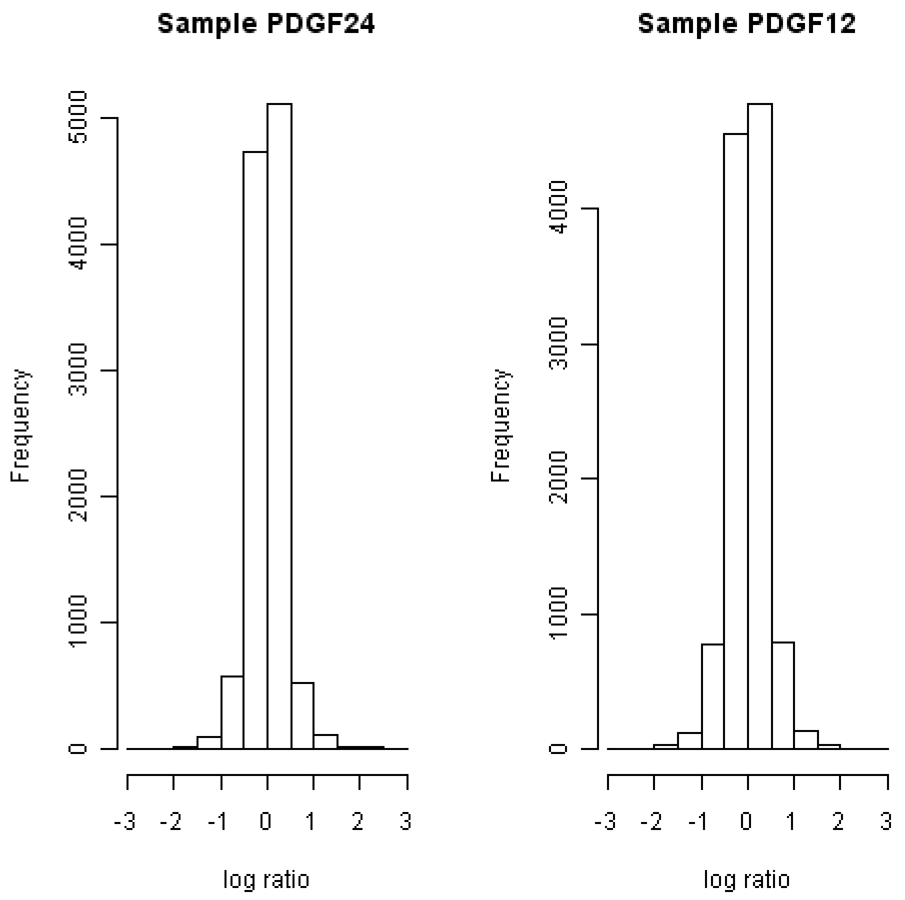

Figure S1. Histograms of log ratios of expression between experimental samples and control samples
